# The effect of genetic variations in the choline acetyltransferase gene (ChAT) on waterpipe tobacco smoking dependence

**DOI:** 10.18332/tid/118233

**Published:** 2020-03-31

**Authors:** Omar F. Khabour, Rawan N. Abu-Eitah, Karem H. Alzoubi, Ahmed Abu-Siniyeh, Thomas Eissenberg

**Affiliations:** 1 Department of Medical Laboratory Sciences, Jordan University of Science and Technology, Irbid, Jordan; 2 Department of Clinical Pharmacy, Jordan University of Science and Technology, Irbid, Jordan; 3 Department of Clinical Laboratory Sciences, College of Applied Medical Sciences, Taif University, Taif, Kingdom of Saudi Arabia; 4 Department of Psychology, Virginia Commonwealth University, Richmond, United States; 5 Center for the Study of Tobacco Products, Virginia Commonwealth University, Richmond, United States

**Keywords:** tobacco, waterpipe, choline acetyltransferase gene, dependence, SNP

## Abstract

**INTRODUCTION:**

Waterpipe tobacco smoking (WS) is a popular form of tobacco use, globally. While the impact of genetic variations on smoking behavior has been well-investigated, few studies have examined this issue with regard to WS. In the current study, associations between choline acetyltransferase gene (ChAT) rs1917810 and rs7094248 polymorphisms and WS dependence were examined.

**METHODS:**

Genotyping of rs1917810 and rs7094248 in 266 Jordanian waterpipe smokers was performed using RFLP-PCR. Dependence on WS was measured using the LWDS-10J scale.

**RESULTS:**

The frequency of rs1917810 G allele was 38.5% and that of the rs7094248 G allele was 40.8%. The rs1917810 was significantly associated with the WS dependence level (p<0.05). Carriers of the AA genotype of rs1917810 were more dependent on WS than those with GG genotype (p<0.05). However, no association between rs7094248 single-nucleotide polymorphism (SNP) and dependence on WS was observed (p>0.05).

**CONCLUSIONS:**

The rs1917810 in the ChAT gene might be associated with dependence on WS.

## INTRODUCTION

Tobacco smoking is a leading cause of preventable diseases and death worldwide^1,2^. More than 6 million people die from tobacco smoking each year, accounting for about 10% of all deaths^3^. Waterpipe tobacco smoking (WS) has become a global form of tobacco use^4,5^, especially among adolescents and young adults^6–8^. For example, in Jordan, prevalence estimates of WS exceeded other tobacco use forms, including cigarettes^9,10^. In addition, according to a study conducted on university students in Middle East and North Africa (MENA) countries, Jordan ranked first in WS use^11^. The widespread of WS is due, in part, to its favorable smell and taste, the social settings in which it is used commonly, and the misconception that WS is less harmful than other tobacco use forms^12^.

When WS is compared to cigarette smoking, both forms share a similar profile of toxicants and carcinogens^13,14^. In addition, both forms increase blood nicotine, which upregulates nicotine receptors in the reward centers of the brain, setting up a potential for nicotine addiction^15,16^.

Describing any genetic influence on smoking behavior would improve understanding of the etiology of tobacco dependence and potentially influence tobacco cessation strategies^17^. Previous studies support the relation between the cholinergic system and tobacco/nicotine dependence^18–21^. Choline acetyltransferase is an enzyme encoded by the ChAT gene and is responsible for synthesis of acetyl choline^22^. Several variations in the ChAT gene have been found to be related to smoking behavior^19,23^. Among such variations is the rs1917810 single-nucleotide polymorphism (SNP) that has been shown to be associated with smoking cessation^19^.

Findings of previous studies on cigarette smoking have identified different genetic variants associated with smoking behavior phenotypes, including initiation, dependence, and cessation^24–27^. Studies examining the influence of genetic variation on WS behavior are lacking. Therefore, in the current study, we aimed to study the effect of rs7094248 and rs1917810 ChAT SNPs on WS dependence in a sample of exclusive waterpipe smokers from Jordan.

## METHODS

### Study participants

The study design was cross-sectional and investigated genetic variations that contribute to waterpipe smoking behavior. Participants were Arab Jordanian adults who were ‘current’ waterpipe smokers and who reported that they smoked tobacco in a waterpipe exclusively (i.e. no cigarette or any other smoking types, including e-cigarettes). Participants were recruited from universities, cafes and restaurants from Central and Northern regions of Jordan. Exclusion criteria were any tobacco use other than WS. A total of 266 males and females, who qualified, participated by completing a questionnaire and donating a blood sample. The Institutional Review Board (IRB) at Jordan University of Science and Technology approved the study (Approval Number: 195/2012). Written informed consent was collected from all participants, as required by the IRB.

### Waterpipe data collection

A previously validated questionnaire was used to collect sociodemographic characteristics and waterpipe tobacco use among participants^28–30^. The questionnaire included the 10 items of the Lebanese Waterpipe Dependence Scale that has been validated in Jordan (LWDS-10J)^26^ and used to estimate and measure WS dependence level. Based on a threshold score of 10, smokers were categorized^30^ into: mild smokers with LWDS-10J score ≤10; moderate smokers with LWDS-10J score >10 through ≤20; and heavy smokers with LWDS-10J score >20. The questionnaire also asked about ‘quit attempt’, which is defined in the current study as stopping the use of waterpipe for a period with an intention to quit smoking.

### Blood samples and DNA extraction

About 3 mL of venous blood were collected from each participant in EDTA vacutainer tubes. DNA was extracted from blood samples using Wizard Genomic DNA Purification Kit (Promega) according to the standard manufacturer instructions. Concentration and quality of extracted DNA were checked and then samples were stored at −20 °C until used^31^.

### Genotyping of rs1917810 and rs7094248 SNPs in ChAT gene

Genotyping of rs1917810 and rs7094248 SNPs in ChAT gene was performed by using standard PCR, followed by restriction fragment length polymorphism (PCR-RFLP) technique. Target DNA was amplified using the forward primer 5’-TGAGCCTGCTAAGGGGTCTA-3’ and the reverse 5’-GAGCCCAGGGAACAAGAGAT-3’ for rs1917810, and forward primer 5’-TTGCTCACCAAGCAAGACAG-3’ and reverse 5’-GTCCTTGATGTTGGGAGTGG-3’ for rs7094248. The PCR conditions were as previously described^19^. The 152 bp amplification product was digested at 37 °C overnight by *XmnI* restriction enzyme (New England Biolabs, Frankfurt, Germany). On the other hand, the 159 bp amplification product for rs1917810 was digested at 37 °C overnight by *TfiI*restriction enzyme (New England Biolabs, Frankfurt, Germany).

### Statistical analysis

SPSS version 22.0 (Inc., Chicago, IL) was used for data analysis using the statistical package for the social sciences. Chi-squared test was used for: 1) comparing genotype and allele frequencies, and 2) testing whether individual variants were in Hardy-Weinberg equilibrium (HWE) at each locus in the samples. One-way ANOVA, followed by Tukey’s post hoc test, was used to compare addiction scores according to genotype. A p<0.025 was used to indicate significant difference between groups.

## RESULTS

Sociodemographic characteristics of the 266 participants (Table 1) showed that 53.6% of the participants were men, and mean age was 29.2 years (SD=9.8, range 18–62 years). About half of the sample (45.8%) reported a household income between 501–1100 JOD (1 JOD about 1.41 US$), which reflects the average household income in Jordan (approximately 550 JOD, Jordanian Dinar).

**Table 1:** Participant sociodemographic characteristics, Jordan (N=266)

*Variables*	*Waterpipe smokers n (%)*
**Age** (years), mean ± SD	29.2 ± 9.8
18–29	159 (59.8)
30–39	64 (24.1)
≥40	43 (16.2)
**Gender**	
Male	143 (53.6)
Female	121 (45.3)
**Monthly household income** (JOD)	
<500	35 (13.1)
501–1100	122 (45.8)
>1100	109 (40.8)

JOD: 1 Jordanian Dinar about 1.41 US$.

About 40.5% of the participants started WS during school age (Table 2). Among participants, 47.2% reported daily WS, while weekly was reported by 37.1%, and monthly by 15.4%. Regarding duration of WS session, 37.1% of participants spend less than 60 minutes per session, and about one-third (32.6%) spend between 1–2 hours. The majority (53.9%) smoked one head per session. About 74.8% owned a waterpipe and 62.5% took it to places that did not offer waterpipe. No more than 44.4% of participants reported a WS quit attempt (Table 2).

**Table 2:** Waterpipe smoking characteristics of participants, Jordan (N=266)

*Variables*	*Waterpipe smokers n (%)*
**Age of initiation waterpipe smoking** (years)	
≤14 (middle school range)	32 (12.0)
15–17 (high school range)	76 (28.5)
18–21 (university range)	82 (30.8)
>21	76 (28.5)
**Duration of smoking since initiation** (years), mean ± SD	9.57 ± 3.8
**Frequency of smoking**	
Daily	126 (47.2)
Weekly	99 (37.1)
Monthly	41 (15.4)
**Waterpipe session duration** (hours)	
<1	99 (37.1)
1–2	87 (32.6)
>2	80 (30)
**Number of tobacco heads per session**	
1	144 (53.9)
2	71 (26.6)
≥3	51 (19.2)
**Owned waterpipe device**	199 (74.8)
**Took waterpipe to places that did not provide it** (if owned)	167 (62.5)
**Made quit attempts**	118 (44.4)

Table 3 shows the genotypes and allele frequencies of rs1917810 and rs7094248 SNPs and level of addiction based on LWDS-10J dependence categories (heavy, moderate, mild). The rs1917810 SNP genotypes are associated with waterpipe dependence (p<0.05). The frequency of AA genotype of the rs1917810 SNP was higher in heavy smokers, whereas the GG genotype frequency was higher in mild smokers. When allele frequencies were considered, the A allele was related mainly to heavy smokers and G allele to mild smokers (p<0.01). With respect to rs7094248 SNP, no significant association was found with waterpipe dependence (p>0.05) (Table 3).

**Table 3:** Genotype and allele frequencies of rs1917810 and rs7094248 by waterpipe smoking dependence level, among adults, Jordan (N=266)

*rs1917810*				

	*Dependence level (N=266), n (%)*			*Chi-squared statistics*

*Genotype*	*Heavy (23)*	*Moderate (83)*	*Mild (160)*	
**A/A**	14 (14.3)	35 (35.7)	49 (50.0)	χ^2^ (2, N=266) = 12.89 p=0.0118
**A/G**	7 (5.3)	42 (32.1)	82 (62.6)	
**G/G**	2 (5.4)	6 (16.2)	29 (78.4)	
**Allele A**	35 (10.7)	112 (34.3)	180 (55.0)	χ^2^ (2, N=532) = 10.35 p=0.0056
**Allele G**	11 (5.4)	54 (26.3)	140 (68.3)

**Table:** 

*rs7094248*				

	*Dependence level (N=266), n (%)*			*Chi-squared statistics*

*Genotype*	*Heavy (23)*	*Moderate (83)*	*Mild (160)*	
**C/C**	8 (8.04)	27 (29.02)	58 (55.94)	χ^2^ (2, N=266) = 5.17 p=0.269
**C/G**	9 (11.15)	47 (40.25)	73 (77.59)	
**G/G**	6 (3.80)	9 (13.73)	29 (26.47)	
**Allele C**	25 (27.24)	101 (98.29)	189 (189.47)	χ^2^ (2, N=532) = 0.636 p=0.727
**Allele G**	21 (18.76)	65 (67.71)	131 (130.53)	

The association between quit attempts of WS and rs1917810 and rs7094248 SNPs is shown in Table 4. A significant association between quit attempts and rs1917810 genotypes was detected (p<0.05). The frequency of AA was lower in individuals who made quit attempts than those who did not (34.7% versus 65.3%, respectively). In addition, when alleles were considered, the A allele was found to be associated with fewer quit attempts (p<0.05). With respect to rs7094248 SNP, no significant association was found with quit attempts (p>0.05) (Table 4).

**Table 4:** Genotype of rs1917810 and rs7094248 among waterpipe smokers with respect to quit attempts among adults, Jordan (N=266)

*SNP*		*Made quit attempts*		

		*Yes*	*No*	*Chisquared statistics*
**rs1917810 Genotype**	**A/A**	34 (34.7)	64 (65.3)	χ^2^ (2, N=266) = 5.98 p=0.050
	**A/G**	66 (50.4)	65 (49.6)	
	**G/G**	18 (48.7)	19 (51.3)	
**rs1917810 alleles**	**Allele A**	134 (41)	193 (59)	χ^2^ (1, N=532) = 3.93 p=0.047
	**Allele G**	102 (49.8)	103 (50.2)	
**rs7094248 Genotypes**	**C/C**	40 (43.0)	53 (57.0)	χ^2^ (2, N=266) = 0.523 p=0.769
	**C/G**	60 (46.5)	69 (53.5)	
	**G/G**	18 (40.9)	26 (59.1)	
**rs7094248 alleles**	**Allele C**	140 (44.4)	175 (55.6)	χ^2^ (1, N=532) = 0.002 p=0.963
	**Allele G**	96 (44.2)	121 (55.8)	

## DISCUSSION

This study examined the association of rs1917810 and rs7094248 SNPs in the ChAT gene with WS dependence, in a sample of exclusive waterpipe smokers. The results showed an association between rs1917810 SNP and WS dependence and quitting. Results of previous studies on cigarette smoking have identified different genetic variants among genes incorporated with several neuronal pathways associated with smoking behavior phenotypes, including initiation, dependence, and cessation^24–27^. Nicotine is associated with tobacco dependence by affecting the release of different neurotransmitters including acetylcholine and dopamine in the central reward pathway of the brain^23,25^. Choline acetyltransferase enzyme is an important player in cholinergic neurons^32^ and nicotine modulates its expression and activity^23^. The ChAT gene was suggested as a biologically plausible candidate to be associated with smoking dependence and smoking behavior.

The results of the current study showed a statistically significant association between the rs1917810 SNP and WS dependence, as measured using LWDS-10J. In addition, the rs1917810 SNP was associated with quitting. This is consistent with the study of Ray et al.^19^ that reported evidence of a convergent association between ChAT rs1917810 SNP and both smoking cessation and nicotine dependence. The present findings suggest that a significant genotypic difference exists at the three levels of addiction: heavy, moderate, and mild. The minor allele G being expressed more highly than the A allele in mild smokers, presenting a lower addiction level than that found with A allele. This result supports the notion that genetic variation in the ChAT gene contributes to the level of WS dependence and quitting. However, a previous study^23^ reported no significant association between nicotine dependence and rs1917810 SNP. With respect to rs7094248, the results showed a lack of association between this SNP and WS dependence. This is consistent with a previous study that reported that rs7094248 was not associated with smoking dependence among treatment-seeking smokers of European ancestry^19^.

### Limitations

Among the limitations of the current study is the relatively small sample size, a result of our attempt to include individuals who smoked tobacco with a waterpipe only. Additional studies with larger sample sizes are needed, though the same challenge remains, and these studies might also require additional ChAT SNPs. Pharmacogenetic studies are also recommended to examine the impact of rs1917810 SNP on WS treatment. Moreover, determining the generalizability of the results reported here may require replication using participants across various world regions. Finally, covariates related to smoking habits or dependence, such as education, socioeconomic level and exposure to secondhand smoke etc., might affect the observed genetic association with waterpipe smoking behavior. Thus, it is recommended that such confounders are considered in future studies.

## CONCLUSIONS

Individual SNP association analysis revealed that ChAT is associated with WS behavior that includes dependence and quit attempts, making ChAT a suitable target for additional research on the mechanisms underlying association with WS dependence.
